# Efficacy and Safety of Magnetic Resonance‐Guided Focused Ultrasound Thalamotomy in Essential Tremor: A Systematic Review and Metanalysis

**DOI:** 10.1002/mds.30188

**Published:** 2025-04-17

**Authors:** Alyssa Shiramba, Steven Lane, Nicola Ray, Tom Gilbertson, Rajesha Srinivasaiah, Jay Panicker, Mark Radon, Jibril Osman‐Farah, Antonella Macerollo

**Affiliations:** ^1^ School of Medicine University of Liverpool Liverpool UK; ^2^ The Walton Centre NHS Foundation Trust for Neurology and Neurosurgery Liverpool UK; ^3^ Institute of Data Health Sciences University of Liverpool Liverpool UK; ^4^ Department of Psychology Manchester Metropolitan University Manchester UK; ^5^ Department of Neurology Ninewells Hospital & Medical School Dundee UK; ^6^ Division of Imaging Science and Technology Medical School, University of Dundee Dundee UK; ^7^ Institute of Systems, Molecular and Integrative Biology The University of Liverpool Liverpool UK

**Keywords:** MRI‐guided focused ultrasound, essential tremor, safety, efficacy, tremor recurrance

## Abstract

**Background:**

Magnetic resonance‐guided focused ultrasound (MRgFUS) is an established surgical treatment for essential tremor, providing tremor relief without the need for an incision or general anesthesia. Meta‐analyses have been limited in their exploration of the durability of the treatment effect.

**Objectives:**

The study aimed to assess the treatment effect and safety of this procedure over time. Different to other meta‐analyses, this study assessed the durability of efficacy over time from 1 month to 5 years follow‐up. Investigating the recurrence of tremor was an important target of this work.

**Methods:**

A systematic search of the literature utilizing set search criteria was conducted with the PubMed, Scopus, Web of Science, and Cochrane library databases, in accordance with the Preferred Reporting Items for Systematic Reviews and Meta‐Analyses (PRISMA) guidelines. Data analysis was conducted in R, utilizing a random‐effects model for meta‐analysis and a mixed‐effects model for meta‐regression.

**Results:**

Forty‐five studies met the inclusion criteria, of which 42 were included in the analyses. Significant changes in hand tremor, total tremor, disability scores, and quality of life scores were demonstrated across the time points investigated, the pooled standardized mean differences being −2.36 (*P* < 0.0001), −2.08 (*P* < 0.0001), −2.85 (*P* < 0.0001), and −1.41 (*P* < 0.0001) 1 year post‐operation. Sensory symptoms and unsteadiness adverse events were frequently observed, with pooled proportions of 22% (95% CI 15%; 31%) and 23% (95% CI 16%; 31%) 1 month post‐MRgFUS.

**Conclusion:**

Although the procedure demonstrated efficacy and safety across the studies evaluated, meta‐regression analysis suggests a decrease in treatment effect over time that requires further investigation. © 2025 The Author(s). *Movement Disorders* published by Wiley Periodicals LLC on behalf of International Parkinson and Movement Disorder Society.

Essential tremor (ET) is a chronic movement disorder, defined as a “bilateral upper limb action tremor” by the International Parkinson and Movement Disorder Society.^1^ ET becomes more prevalent with age and significantly impacts quality of life (QOL).^2^, ^3^

Published meta‐analyses on the use of magnetic resonance‐guided focused ultrasound (MRgFUS) in ET have demonstrated its safety and efficacy at various time points. Despite this, there has been limited investigation into the impact of time on hand tremor scores.^4^, ^5^, ^6^ One meta‐analysis investigated this via meta‐regression; however, the impact of time on tremor scores was not statistically significant.^5^ As such, the primary objective of this meta‐analysis with meta‐regression aims to further investigate the hand tremor scores over time, in light of the further studies that have been published since the previous meta‐regression.

MRgFUS offers an alternative in patients with pharmaco‐resistant forms of ET.^7^, ^8^, ^9^ This meta‐analysis aimed to assess the efficacy and safety of MRgFUS through the examination of individual studies which met predetermined eligibility criteria. The outcome measures, tremor scores, disability scores, QOL scores, intraoperative adverse events (AEs), and postoperative AEs, across follow‐up intervals of 1 month, 3 months, 6 months, and 1 year, were used to assess the efficacy and safety of MRgFUS as well as the duration of the tremor's improvement in the postoperative period.

The Clinical Rating Scale for Tremor (CRST) is widely used to assess tremor severity, consisting of a total score, as well as Parts A, B, and C.^10^ Part A and B scores for the treated hand are commonly combined to produce an “AB” or “Hand tremor” score. QOL is often determined via the disability score (as assessed via Part C of the CRST) and/or Quality of Life in Essential Tremor Questionnaire (QUEST) scores.^11^

## 1 Methods

### 1.1 Literature Search

The databases used to identify relevant studies for inclusion were PubMed, Scopus, Web of Science, and Cochrane Library. There were no limitations in terms of publication date, but only English language articles were exported from the databases. Key terms searched included variations of “MRIgFUS,” “MRgFUS,” “focused ultrasound,” “HIFU,” “ultrasound,” “thalamotomy,” “high intensity focused ultrasound ablation,” and “essential tremor”; the search was run to April 2024. The search strings are presented in Table S1.

### 1.2 Study Eligibility

**FIG. 1:**
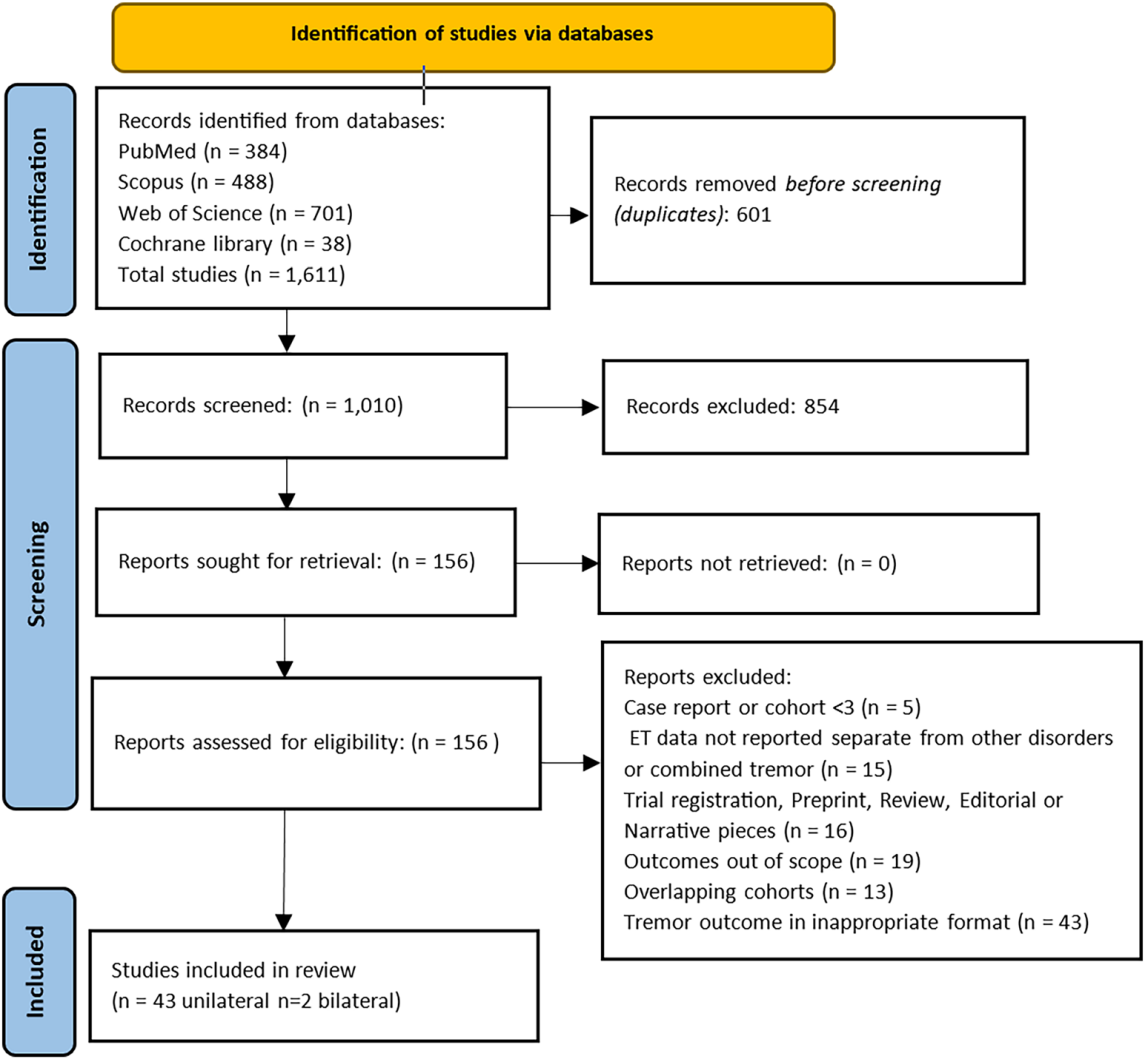
Preferred Reporting Items for Systematic Reviews and Meta‐Analyses (PRISMA) flow diagram of screening and selection process. This demonstrates the screening and selection process. Forty‐five studies met the inclusion criteria, of which two were studies of bilateral magnetic resonance‐guided focused ultrasound (MRgFUS). Forty‐two studies were ultimately included in the meta‐analyses. ET, essential tremor. [Color figure can be viewed at wileyonlinelibrary.com]

Preferred Reporting Items for Systematic Reviews and Meta‐Analysis Protocols (PRISMA‐P) were used as a guideline throughout the screening process, and the results are displayed in Figure 1.^12^ Inclusion criteria were as follows:

1. Data must be reported for ET patients undergoing MRgFUS separately from other conditions within the cohort.
2. Data must present either total CRST scores, hand tremor scores, disability scores, or QUEST scores.
3. Data must be provided for consistent follow‐up intervals and as mean ± SD or as individual participants’ scores whereby mean ± SD can be calculated.
4. Cohorts must consist of at least three patients.

Papers were also excluded if participants received an additional surgical intervention within the study, or participants were comorbid with other movement disorders, and if the publication was a case study or literature review.

After exporting the results from the previously mentioned databases, duplicates were removed, and the subsequent results then underwent title/abstract and full‐text screening according to the inclusion/exclusion criteria outlined above.

### 1.3 Data Extraction

The following data from the final list of included papers were extracted: total CRST scores, hand tremor scores, disability scores, QUEST scores, proportion of intraoperative AEs, and proportions of postoperative AEs. Overlapping or duplicate cohorts were determined by comparing trial registration numbers, study characteristics, and data, if not explicitly reported by the study.

### 1.4 Statistical Analysis

Meta‐analysis was conducted in R using the “meta” (Version 6.5–0) and “esc” packages.^13^, ^14^, ^15^ Means ± standard deviations (SD) for tremor, disability, and QUEST outcomes were used to calculate the standardized mean differences (SMDs) between baseline (pre‐operation) and postoperative scores at 1 month, 3 months, 6 months, and 1 year post‐MRgFUS for each study. A pooled effect size was then calculated. A random‐effects model was utilized to conduct the meta‐analysis of SMDs. A meta‐analysis of proportions was conducted for AEs at the following time points: intraoperative, 1 month, 3 months, 6 months, and 1 year post‐MRgFUS. A random‐effects model was used for this meta‐analysis. Meta‐regression of hand tremor scores was conducted with time as a moderator, including studies reporting hand tremor scores from 1 month to 5 years post‐MRgFUS, utilizing a mixed‐effects model. A *P‐*value of <0.05 was considered statistically significant.

## 2 Results

The results of the screening and selection process are described in Figure 1. There were 45 eligible studies in total identified, of which 42 were included in the meta‐analyses. Chang et al. was excluded from analyses as the relevant cohort data had been reported by other studies in this analysis.^16^ Two studies were assessing bilateral procedures, and the remainder were unilateral procedures. The bilateral MRgFUS studies were not included in this meta‐analysis due to their substantial inherent differences from unilateral treatment, such as differences in the way the procedure is conducted, varying tremor outcomes, and side effects.^17^, ^18^ Moreover, as there were only a small number of bilateral studies identified, a separate analysis of bilateral procedures was not possible. Another three studies were not included in the meta‐analysis due to overlaps with other study cohorts, with data that were not reported separately.^19^, ^20^, ^21^

### 2.1 Study Characteristics, Patient Demographics, and Intervention Parameters

Studies included spanned over a decade, from 2013 to 2024, with sample sizes ranging from 4 to 101 patients. The longest period of follow up was 5 years, as reported by Cosgrove et al.^22^ Four of the included studies were prospective reports on the follow‐up of patients from the randomized controlled trial (RCT) cohort in Elias et al.^16^, ^22^, ^23^, ^24^, ^25^ This RCT is separated in meta‐analysis plots into patients who were initially randomized to receive the procedure (“Thalamotomy”), those who crossed over to the intervention group later in the study (“Crossover”), or were re‐treated (“Crossover & Retreat”). Purrer et al. separated their cohort into those who did or did not have gustatory side effects (“GUST” and “NO‐GUST”); scores were presented separately for these groups.^26^ All but one study were non‐RCTs, with the remainder utilizing before–after study designs, wherein the same cohort pre‐intervention is used as a control or comparator.

The average cohort ages were reflective of the typical ET population; cohorts largely consisted of elderly patients, with the exception of Wu et al., in which the mean age was the lowest of all the included studies at 59.14 ± 13.5 years.^27^ The majority of patients were male, Skull density ration (SDR) varied from a mean of 0.34 to 0.56, total sonications ranged from a mean of 7.2 to 22.5, mean maximum energy 10,320 J to 26,867 J, and mean peak temperature 53°C to 63.1°C. Study, intervention, and patient characteristics are described in Tables 1 and 2. The most common anatomical target was the ventral intermediate nucleus (VIM), whilst the dentato‐rubro‐thalamic tract (DRTT) and cerebellothalamic tract (CTT) were also treatment targets in two studies each, respectively.

**TABLE 1:** Study and participant characteristics

Reference	Year	First author	Study design	Cohort size	Age (years)	Proportion male
^39^	2013	Elias	NS	15	66.6 ± 8.0	67%
^40^	2013	Lipsman	NS	4	70.8	100%
^41^	2015	Chang	NS	8	66.1 ± 5.6	7 M, 1 F
^23^	2016	Elias	RCT	Thalamotomy: 56	Thalamotomy: 70.8 ± 8.7	Thalamotomy: 66%
				Crossover: 21		
^42^	2016	Gallay	NS	21	69.1 ± 9.2	15 M, 6 F
^43^	2017	Schreglmann	P	6	70.7 ± 8.5	
^44^	2018	Boutet	R	66	72.4 ± 8.4	71%
^16^	2018	Chang^a^ (continuation of Elias et al.)	P	76	71.0 ± 8.3	68%
^45^	2018	Federau	R	7	78 ± 6	5 M, 2 F
^46^	2018	Harary	NS	7	67.7 ± 6.3	5 M, 2 F
^47^	2018	Iacopino	NS	13	65.22 ± 11.87	10 M, 3 F
^48^	2018	Jung	P	20	64.1	17 M, 3 F
^49^	2018	Tian	R	8		
^50^	2018	Zaaroor	NS	18	73.1 ± 6.2	12 M, 6 F
^51^	2019	Gasca‐Salas	NA	23	64.1 ± 14.16	17 M, 6 F
^24^	2019	Halpern (continuation of Elias et al.)	P	75	71 ± 8.3	
				52 at 3 years FU		
^52^	2019	Krishna	P	10	70.8 ± 9.7	60%
^53^	2019	Miller	R	4		
^25^	2019	Park (continuation of Elias et al.)	NS	12 at 4 years FU	61.7 ± 8.1	10 M, 2 F
^54^	2019	Pineda‐Pardo	P	24	68.0 ± 10.1	17 M, 7 F
^55^	2020	Jones	P	12	72.2 ± 7.8	8 M, 4 F
^32^	2020	Fukutome	R	15	62.9 ± 11.3	11 M, 4 F
^56^	2021	Chang	P	6	Whole cohort (6 ET and 2 PD): 68.87	
^57^	2021	Mazerolle	NA	17	73.5 ± 8.8	15 M, 2 F
^58^	2021	Tommasino	R	30	66 ± 11.84	24 M, 6 F
^27^	2021	Wu	R	48	59.14 ± 13.5	31 M, 17 F
^59^	2022	Lu	NS	30	61.97 ± 10.77	21 M, 9 F
^60^	2022	Pohl	NS	15	66.2 ± 15.4	12 M, 3 F
^38^	2022	Purrer	NS	37	69.4 ± 12.2	25 M
^61^	2022	Tani	R	7	70.7 ± 6.1	5 M, 2 F
^22^	2022	Cosgrove (continuation of Elias et al.)	P	Start: 75	At 5 years FU: 75 ± 8.4	At 5 years FU: 30 M, 10 F
				At 5 years FU: 40		
^62^	2023	Kato	NS	15	72.8 ± 5.39	11 M, 4 F
^63^	2023	Lueckel	NS	18	71.44	14 M, 4 F
^64^	2023	Pae	R	85	65.3 ± 8.7	67 M, 18 F
^26^	2023	Purrer	NS	26	GUST: 70.8 ± 8.7	GUST: 8
				13 patients with gustatory side effects (GUST) and 13 patients without gustatory side effects (NO‐GUST) analyzed in this study	NO‐GUST: 66.6 ± 12.8	NO‐GUST: 11
^65^	2023	Sastre‐Bataller	NS	32 (24 in tremor analysis)		18 M, 14 F
^66^	2023	Wang	R	27	61.15 ± 11.21	19 M, 8 F
^28^	2023	Perez‐Garcia	R	43		21 M, 22 F
^67^	2023	Saporito	P	22	69.5 ± 10.0	Whole sample (22 ET + 18 PD patients): 38 M, 2 F
^29^	2024	Gurgone	NS	10	71 ± 7.8	6 M, 4 F
^33^	2024	Zong	P	10	65.2 ± 5.2	7 M, 3 F
^30^	2024	Ito	P	10 (6 at 5 years FU)	67.1 ± 17.5	8 M, 2 F
^31^	2024	Hino	R	101	70 ± 12	74 M

*Note*: Details of included unilateral magnetic resonance‐guided focused ultrasound (MRgFUS) papers, including year, first author, cohort size, mean participant age, and the proportion of male participants (% refers to the percentage male).

Abbreviations: NS, not specified; M, male; F, female; RCT, randomized controlled trial; P, prospective; R, retrospective; ET, essential tremor; PD, Parkinson's disease; NA, not available; FU, follow‐up.

^a^ Chang et al. was not included in the analyses as the relevant outcomes for this cohort have already been reported by other studies.

**TABLE 2:** Summary of intervention parameters

Reference	Year	First author	Anatomical target	Skull density ratio	Sonications (n)	Maximum energy delivered (J)	Peak temperature (°C)
^39^	2013	Elias	VIM		17.9 ± 4.6	10,320 ± 4537	58.5 ± 2.5
^40^	2013	Lipsman	VIM		22.5		59.3
^41^	2015	Chang	VIM				53 ± 3.3
^23^	2016	Elias	VIM		Thalamotomy: 18.5 ± 5.2	Thalamotomy: 14,497.0 ± 6695.7	Thalamotomy: 55.6 ± 2.3
^42^	2016	Gallay	CTT			16,073 ± 6037	
^43^	2017	Schreglmann	CTT		11 ± 3.2	12,008 ± 4441	62.0 ± 2.5
^44^	2018	Boutet	VIM	0.48 ± 0.1			56.6 ± 2.3
^16^	2018	Chang (continuation of Elias et al.)	VIM		18.5 ± 5.2		55.6 ± 2.3
^45^	2018	Federau	VIM		18.6 ± 5.7		
^46^	2018	Harary	VIM				
^47^	2018	Iacopino	VIM				
^48^	2018	Jung	VIM		16.8	15,910 ± 5702.7	57.9
^49^	2018	Tian	VIM				
^50^	2018	Zaaroor	VIM		20.9 ± 6.4	12,232 ± 3190	56.9 ± 2.5
^51^	2019	Gasca‐Salas	VIM				
^24^	2019	Halpern (continuation of Elias et al.)	VIM				
^52^	2019	Krishna	VIM	0.54 ± 0.1	13.9 ± 4.5		
^53^	2019	Miller	DRTT				
^25^	2019	Park (continuation of Elias et al.)	VIM	0.49 ± 0.08	17.3 ± 1.6	15,552.4 ± 6574.1	
^54^	2019	Pineda‐Pardo	VIM				
^55^	2020	Jones	VIM	0.49 ± 0.14	12.67 ± 2.02	26,866.67 ± 18,275.14	55.1 ± 2.4
^32^	2020	Fukutome	VIM	0.45 ± 0.11		16,275 ± 8610	57.3 ± 1.9
^56^	2021	Chang	VIM	0.34	Whole cohort (6 ET and 2 PD): 15		Whole cohort: 55.88
^57^	2021	Mazerolle	VIM	0.54 ± 0.09			
^58^	2021	Tommasino	VIM	0.45 ± 0.072		14,985.37 ± 8421.01	61.07 ± 3.93
^27^	2021	Wu	VIM	0.5 ± 0.1	10.0 ± 2.6	19,710.5 ± 8624.9	57.0 ± 2.4
^59^	2022	Lu	VIM	0.51 ± 0.10			
^60^	2022	Pohl	VIM	0.45 ± 0.1			
^38^	2022	Purrer	VIM		8.6 ± 3.5		62.4 ± 3.6
^61^	2022	Tani	VIM				
^22^	2022	Cosgrove 4‐ and 5‐year FU (continuation of Elias et al.)	VIM				
^62^	2023	Kato	VIM				
^63^	2023	Lueckel	VIM	0.46			
^64^	2023	Pae	VIM	0.54 ± 0.08	14.1 ± 2.3	20,166.6 ± 7885.8	57.2 ± 2.0
^26^	2023	Purrer	VIM	GUST: 0.44 ± 0.07	GUST: 8.0 ± 2.7		GUST: 63.1 ± 4.1
				NO‐GUST: 0.48 ± 0.10	NO‐GUST: 7.2 ± 2.2		NO‐GUST: 62.0 ± 3.3
^65^	2023	Sastre‐Bataller	VIM	0.56 ± 0.08	8.03 ± 2.91	12,185.94 ± 6506.49	58.56 ± 2.20
^66^	2023	Wang	VIM				
^28^	2023	Perez‐Garcia	Intersection of decussating and non‐decussating DRTT	0.52 ± 0.09		11,727 ± 7214	
^67^	2023	Saporito	VIM	Whole sample (22 ET + 18 PD patients): 0.43 ± 0.07	Whole sample (22 ET + 18 PD patients): 11.06 ± 3.87		
^29^	2024	Gurgone	VIM				
^33^	2024	Zong	VIM	0.49 ± 0.09			
^30^	2024	Ito	VIM	0.39 ± 0.09	9.7 ± 1.6	27,585.2 ± 9127.8	57 ± 1.8
^31^	2024	Hino	VIM	0.4 ± 0.1	8.8 ± 2.4	23,300 ± 10,900	54.1 ± 3.3

*Note*: A summary table of intervention parameters from unilateral magnetic resonance‐guided focused ultrasound (MRgFUS) studies, including sonication number, peak temperature reached (in degrees Celsius), maximum energy (in Joules), anatomical target, and patient skull density ratio. Metrics are mean or mean ± standard deviation, unless otherwise specified.

Abbreviations: VIM, ventral intermediate nucleus; CTT, cerebellothalamic tract; DRTT, dentato‐rubro‐thalamic tract; ET, essential tremor; PD, Parkinson's disease; FU, follow‐up; GUST, gustatory side effects; NO‐GUST, without gustatory side effects.

### 2.2 Tremor Reduction

Improvement in tremor symptoms were determined via total CRST scores and CRST hand tremor scores. Changes in these scores from baseline to 1 month, 3 months, 6 months, and 1 year were assessed via standardized mean differences, of which the pooled values are reported in Table S2 alongside measures of variance and statistical significance. Separate analyses were conducted for each of these time points. Negative values represent a decline in tremor scores, which demonstrate an improvement in tremor.

#### 2.2.1 Total CRST Scores

Total tremor scores at baseline versus 1 month, 3 months, 6 months, and 1 year post‐operation demonstrated improvements in all studies, with results depicted in Figure S1. Zong et al. demonstrated the greatest improvement in scores post‐MRgFUS across all time points assessed in this analysis.^33^ The pooled SMDs of 14 studies reporting the total tremor score 1 month post‐MRgFUS was −2.54 (95% CI: −3.00; −2.09, *P* < 0.0001). This suggests a significant reduction in tremor 1 month after MRgFUS. This effect size was greater than that calculated for 3 months, 6 months, and 1 year post‐operation. The pooled SMDs of 14 studies reporting total tremor scores at 3 months post‐MRgFUS was −1.87 (95% CI: −2.22; −1.53, *P* < 0.0001). At 6 months, the pooled SMDs of 16 studies was −2.19 (95% CI: −2.57; −1.82, *P* < 0.0001). A reduction in tremor was also demonstrated in the 11 studies reporting total tremor scores 1 year post‐operation, with a pooled SMD of −2.08 (95% CI −2.52; −1.63, *P* < 0.0001). Overall, all pooled effect sizes for the aforementioned time points suggest a treatment benefit of MRgFUS. However, studies displayed high heterogeneity, with *I* ^2^ values ranging from 68% to 82%.

#### 2.2.2 Hand Tremor Scores

**FIG. 2:**
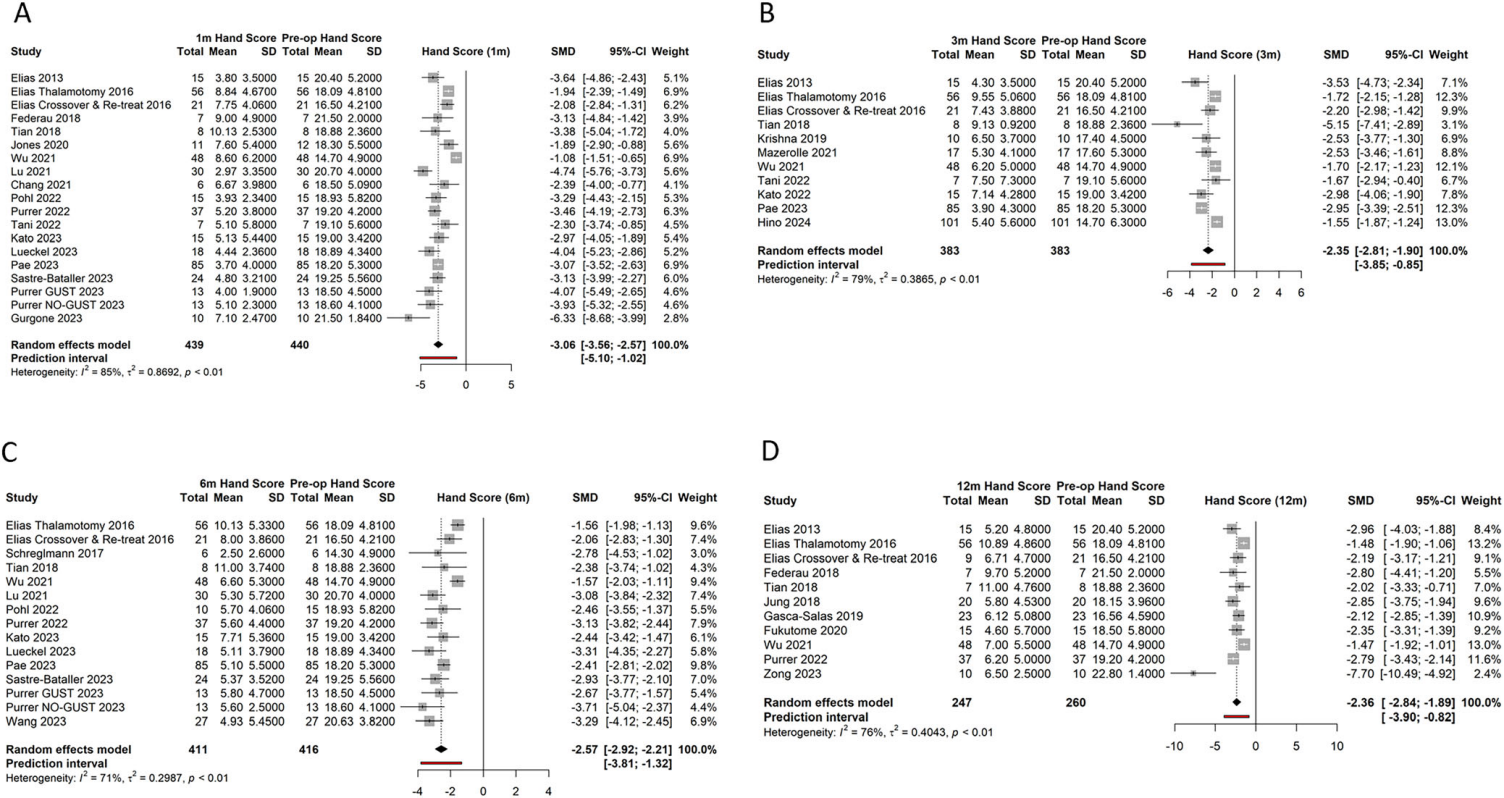
Hand tremor score forest plots. Forest plots (A–D) depict the mean scores and standard deviations pre‐magnetic resonance‐guided focused ultrasound (MRgFUS) and post‐MRgFUS, standardized mean differences (SMDs), pooled SMDs, and measures of heterogeneity for hand tremor scores 1 month (A), 3 months (B), 6 months (C), and 1 year (D) post‐operation. [Color figure can be viewed at wileyonlinelibrary.com]

Hand tremor scores (HTS) at baseline versus 1 month, 3 months, 6 months, and 1 year post‐operation are depicted in Figure 2, with all studies demonstrating an improvement from baseline. The pooled SMD of the 17 studies reporting HTS at 1 month post‐MRgFUS was −3.06 (95% CI: −3.56; −2.57, *P* < 0.0001). At 3 months post‐MRgFUS the pooled SMD of 10 studies was lower at −2.35 (95% CI: −2.81; −1.90, *P* < 0.0001). Efficacy was also demonstrated in 13 studies at 6 months and 10 studies at 1 year post‐MRgFUS, with pooled SMDs of −2.57 (95% CI: −2.92; −2.21, *P* < 0.0001) and −2.36 (95% CI: −2.84; −1.89, *P* < 0.0001), respectively. Of note, Zong et al. demonstrated the greatest SMD (−7.70 at 1 year).^33^ Overall, this suggests a significant reduction in hand tremor at every follow‐up interval including 1 year post‐operation, with the greatest treatment effect being 1 month post‐procedure. The studies showed high heterogeneity, with *I* ^2^ values ranging from 71% to 85%. The results are summarised in Table S2.

**FIG. 3:**
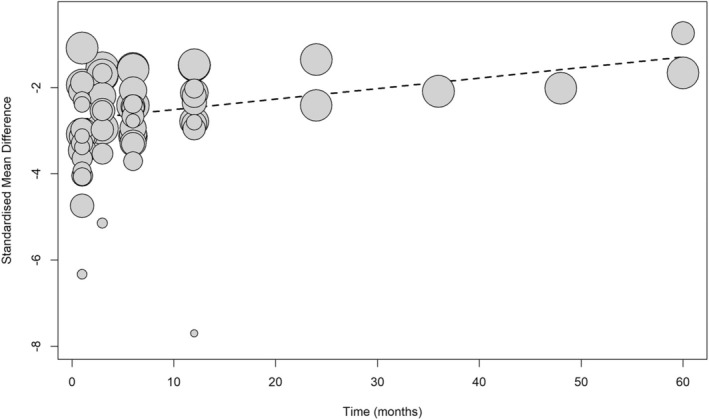
A bubble plot of hand tremor meta‐regression. This bubble plot presents the results of the meta‐regression of hand tremor scores with time in months as the moderator. The trend suggests a significant decrease in effect size with time (*P* = 0.0021).

Meta‐regression, displayed in Figure 3, revealed a statistically significant decrease in hand tremor effect sizes over time, from 1 month to 5 years post‐intervention (*P* = 0.0021). However, time accounted for a small amount of the variability between effect sizes (*R* ^2^ = 14.58%), meaning that there are likely other factors contributing to this result. Only two cohorts were reported at 2 years post‐operation, one cohort at 3 years and 4 years post‐MRgFUS, and two cohorts at 5 years post‐MRgFUS, with all except Cosgrove et al.^22^ (at 2 years post‐operation) having mean differences that demonstrate a less substantial treatment effect than calculated for 1 year post‐procedure.

### 2.3 Other Efficacy Metrics

Disability scores (Part C of the CRST) and QOL (QUEST) were also assessed. Increasingly negative values represent a beneficial effect. Table S3 displays a summary of meta‐analysis results for these outcomes.

#### 2.3.1 Disability Scores

A significant improvement in disability scores was demonstrated, with nine studies reporting this outcome at 1 month resulting in a pooled SMD of −3.05 (95% CI: −3.82; −2.28, *P* < 0.0001). A large effect was also demonstrated at 3, 6, and 12‐months post‐procedure. Nine studies reporting disability scores at 3 months resulted in a pooled SMD of −2.35 (95% CI: −2.91; −1.79, *P* < 0.0001). Pooled SMDs of 10 studies at 6 months and seven studies at 12 months were − 2.52 (95% CI:‐2.94; −2.10, *P* < 0.0001) and − 2.85 (95% CI: −4.04; −1.67, *P* < 0.0001), respectively. Zong et al. displayed the greatest difference, with an effect size of −7.96 from baseline to 1 year post‐operation.^33^ The studies showed high heterogeneity, with *I* ^2^ values ranging from 77% to 90%. The results are depicted in Figure S2.

#### 2.3.2 QOL Scores

QUEST scores improved across follow‐ups, demonstrating the ability of MRgFUS to improve patient QOL. Three studies reported 1 month and 3 month scores, resulting in pooled SMDs of −1.47 (95% CI: −2.26; −0.69, *P* = 0.0002) and − 1.46 (95% CI: −2.11; −0.82, *P* < 0.0001). A large effect was also seen at 6 months in eight studies, −1.72 (95% CI: −2.32; −1.12, *P* < 0.0001). Improvements in QOL were also demonstrated at 1 year, with a pooled SMD of four studies being −1.41 (95% CI: −1.92; −0.90, *P* < 0.0001). The studies and effect sizes demonstrated variation in terms of large prediction intervals and moderate‐to‐high heterogeneity across time points (I^2^ ranging from 51% to 77%); small sample sizes likely contributed. These results are displayed in Figure S3 and Table S3.

### 2.4 Adverse Events

AEs were extracted to provide insight into the safety of MRgFUS. Those of interest included pain, dizziness, nausea/vomiting, sensory, speech, motor/weakness, and cerebellar‐related events. This included side effects observed intraoperatively and postoperatively up to and including 1 year. Figures [Link], [Link] and Table S4 depict meta‐analysis findings for AEs.

#### 2.4.1 Intraoperative

Intraoperatively, AEs were categorized as follows: sensory, head pain, dizziness, nausea and vomiting (N&V). The most common intraoperative AE assessed was head pain at 39%. This included headache, scalp burn, scalp pain, head pain, and head discomfort. Dizziness had the second highest proportion at 33%, including vertigo, dizziness, sensations of tilting, falling or spinning, vestibular symptoms, and light‐headedness. The proportion of nausea and/or vomiting was 29% and sensory AEs 15%. Sensory events included those reported as paresthesia, flushed or warm sensation, sensory disturbance, or sensory‐related. Many of the above categories were resolved post‐procedure; however, sensory events such as paresthesia were also present postoperatively. The studies showed moderate‐to‐high heterogeneity, with results depicted in Figure S4. Other intraoperative side effects reported by studies included pin‐site bleeding, pain, edema, bruising, balance impairment, skull numbness, anxiety, and back pain.

#### 2.4.2 Postoperative

Postoperative AEs were grouped into the following categories: cerebellar, weakness, subjective cerebellar, sensory, and dysarthria. Despite some pooled proportions displaying low heterogeneity (<25%–30%), a random‐effects model was used to account for the variances that arise from grouping AEs that are not reported in a standardized manner. These variances likely contributed to the heterogeneity of studies, which ranged from low to high (*I* ^2^: 0% to 88%).

#### 2.4.3 The 6‐Month Postoperative Period

The most common AEs at 1 month post‐procedure were subjective cerebellar events at 23%, followed by sensory and cerebellar events at 22% and 21%, respectively. Subjective cerebellar events included subjective unsteadiness, subjective imbalance, disequilibrium sensation, subjective ataxia, subjective gait disturbance, subjective instability, and combined subjective and objective ataxia. Sensory events included paresthesia, numbness, dysesthesia, and sensory‐related events. Cerebellar events included those reported as imbalance, dysmetria, ataxia, balance‐related, gait abnormality, or gait disturbance. Other side effects assessed included weakness (grouping both weakness, weakness, or clumsiness and strength‐related events) at 8%, and dysarthria (speech disturbance) at 9%. These results are shown in Figure S5.

These proportions were lower at 3, 6, and 12 months post‐MRgFUS. Additional AEs reported at 1 to 6 months post‐MRgFUS included subjective and objective involuntary movements, taste disturbances, dysphagia, headache, balance/falls, fatigue, and tinnitus.

#### 2.4.4 12 Months Post‐MRgFUS

The pooled proportions of AEs at 1 year post‐MRgFUS were similar to those at the 6‐month time point; the results are described in Figure S8. The most common AE 1 year post‐MRgFUS was sensory events at 18%, followed by cerebellar events at 10%, and weakness at 3%. Additional AEs at this follow‐up interval were taste disturbance, involuntary movements, subjective unsteadiness, disequilibrium sensation, dysarthria, dizziness, and fatigue.

### 2.5 Bilateral MRgFUS

Two bilateral studies (outlined in Table S5) met the inclusion criteria, with sample sizes of 9 and 10 patients, respectively, and follow‐up periods up to 6 months.^17^, ^18^ Tremor and QOL improvements were demonstrated in both studies. HTS improved from baseline to 6 months post‐FUS2 (post‐second‐sided MRgFUS procedure) in Martinez‐Fernandez et al.^18^ (SMD: −2.68, 95% CI: −3.96, −1.41), along with total tremor scores (SMD: −1.35, 95% CI: −2.38, −0.33).

Disability scores from baseline to 3 months post‐FUS2 improved by −1.38 (95% CI: −2.36, −0.40) in Iorio‐Morin et al. and −0.44 (95% CI: −1.38, 0.49) from baseline to 6 months post‐FUS2 in Martinez‐Fernandez et al. (2021). Additionally, QUEST scores improved from baseline to 3 months post‐FUS2 by −1.16 (95% CI: −2.11, −0.21) in Iorio‐Morin et al.^17^

AEs related to FUS2 that remained unresolved by the end of follow‐up included slurred speech, dysphagia, motor neglect, dysgeusia, and facial numbness.

## 3 Discussion

MRgFUS has provided an alternative treatment option for patients with medication‐refractory ET. Unlike deep brain stimulation (DBS), MRgFUS is a non‐invasive procedure that does not require general anesthesia. Due to the incisionless and non‐invasive nature of the procedure, MRgFUS has shown benefit over other surgical interventions such as radiofrequency thalamotomy and DBS, demonstrated through differences in AEs.^34^, ^35^ The purpose of this meta‐analysis was to provide an up‐to‐date assessment of the treatment benefit and safety of this procedure in ET patients, in order to provide a more accurate treatment effect than published previously, by pooling the results of all relevant studies in accordance with the methodology described previously.^4^, ^5^, ^6^

### 3.1 Efficacy

A key study investigating MRgFUS treatment versus a sham procedure was published by Elias et al., which demonstrated the efficacy of this treatment via an RCT.^23^ Several prospective and retrospective studies investigating the procedure in various institutions have been published since, all of which included in this meta‐analysis demonstrated treatment benefit in terms of hand tremor and total tremor scores, disability scores, and QUEST scores. Treatment efficacy was evident both short‐term and long‐term post‐procedure, with effect sizes similar to those reported previously.^4^, ^5^

The meta‐regression suggested a significant decline in treatment effect over time, which had been proposed by other studies but was not statistically significant.^5^ Although this previous meta‐analysis did not demonstrate statistical significance with regards to this, our meta‐regression showed a statistically significant effect of time as a moderator for hand tremor effect sizes plotted from 1 month to 5 years follow‐up. To the best of our knowledge, this is the first time that this aspect of the treatment has been explored. Efficacy over time is important to evaluate as it could impact a patient's willingness to undergo the procedure if efficacy is not deemed to outweigh the complications. It is also important to consider as regard whether a second operation may be needed, if this is feasible, and at what postoperative time point this should be conducted. Despite the downwards trend observed in this analysis, only two cohorts were investigated at 2 years follow‐up, one cohort at 3 years and 4‐years post‐MRgFUS, and two cohorts at 5 years post‐procedure. Therefore, further studies are needed at these time points to determine the durability of the treatment effect long term via longer follow‐up periods.

Efficacy was demonstrated across anatomical targets, including the VIM, CTT, and DRTT, with the majority of procedures ablating the VIM. Other anatomical targets investigated recently include the posterior subthalamic area, which has shown success in ET patients comparable to the typical target (VIM), with varying AEs.^36^ This warrants future investigation and comparison with the traditional targets.

### 3.2 Complications

The safety of MRgFUS was determined through assessment of AEs. This is vital in planning the management of patients in the perioperative period, such as providing adequate analgesia for intraoperative head pain or ensuring physical rehabilitation is available for significant weakness or gait disturbances post‐operation, and for assessing patient eligibility in terms of capacity to recover from complications.^37^

The most prominent side effect intraoperatively was head pain, with sensory disturbances being present both intraoperatively and postoperatively across follow‐up intervals. This work was limited in assessing safety at 2 years and beyond due to a lack of studies at these time points. Further studies with extensive follow‐up periods are required to accurately assess long‐term complication rates. Of note, in the study with the longest follow‐up, namely 5 years in Cosgrove et al., paresthesia, cerebellar events, and weakness were still observed.^22^

### 3.3 Loss to Follow‐up

Loss to follow‐up is a possible risk of bias and cause for distortion of treatment effect when assessing treatment efficacy and safety in this analysis. Thorough inquiry into and reporting of the reasons for loss of patients during follow‐up should be provided in future studies. For example, Purrer et al. reported that three of four patients who did not attend follow‐up did so due to possible diminishing effects.^38^ Lack of patient retention due to decreased or unwanted treatment effects, and a lack of reporting of this, may lead to an overestimation of treatment benefit and missing AE data. It is important that studies ensure those who decline follow‐up appointments are thoroughly investigated and documented.

### 3.4 Bilateral Studies

Studies investigating the bilateral application of MRgFUS also met the inclusion criteria but were not analyzed due to their inherent differences that would exclude them from incorporation into analyses of unilateral MRgFUS; the limited number of bilateral studies hindered a separate bilateral MRgFUS outcome analysis. Both bilateral studies reported improvements in tremor and disability scores; however, sample sizes were small (n = 9 and n = 10), and neither reported follow‐up data of more than 6 months. Bilateral treatment is not routinely offered for the treatment of ET, despite the condition commonly affecting the upper limbs bilaterally. Despite the potential benefit of bilateral treatment in tremor reduction, the risk of AEs is one of the drawbacks that prevent the use of MRgFUS bilaterally and thus this requires further investigation.^17^

### 3.5 Limitations

The vast variations in the way in which AES were reported means that the results of the meta‐analysis of proportions are not conclusive. The quality of this evidence was limited by the self‐reported nature of many AEs, without clinical examinations to determine objectivity. For example, a distinction was made in this analysis between cerebellar events that were subjective in nature. However, these categories were not definitive in that the “cerebellar” category merely attempted to exclude those events explicitly reported as subjective, rather than including events that were specifically objective as evidenced by formal examination. The lack of standardization in AE assessment and reporting is reflected in the varying degrees of heterogeneity demonstrated across AE analyses. Moreover, it is unclear which patient is being counted at each follow‐up, and a single patient could be responsible for numerous AEs. It is therefore difficult to assess the course of an AE across multiple time points.

Furthermore, there was a high degree of heterogeneity demonstrated across outcome analyses attributable in part due to variances in patient demographics, sample sizes, target selection, and discrepancies in outcome scale usage such as varying maximum possible CRST scores, and the commonly omitted handwriting component of the CRST. Despite this, a moderate‐to‐high heterogeneity was also demonstrated in similar works.^4^, ^5^, ^6^ Moreover, to account for these variances, a random‐effects model was utilized in this meta‐analysis.

Additionally, studies with overlapping cohorts were excluded; however, it is still possible that some cohorts and their data overlap, which could affect the accuracy of the results. Bias could have also arisen due to only studies published in the English language being considered eligible. Relevant studies published in other languages were not included and their exclusion may have altered the results.

There were limited data from 2 years follow‐up onwards, which restricted the ability for such data to be included in pooled analyses and impeded thorough long‐term assessments; publication of further work with extensive follow‐up periods is therefore necessary. As such, the results of the meta‐regression should be interpreted with caution, as there were few studies at time points 2 years and beyond.

The decision to consider studies of varying quality was made due to the importance of considering all relevant studies on the topic in view of the limited number of studies and RCTs, specifically. Ideally, all studies would be RCTs to achieve the highest quality; however, only one was an RCT.

Overall, the high degree of variability in the methodology across studies contributes to the high degree of heterogeneity. Variability is expected when pooling results from numerous institutions across the globe, wherein there are bound to be differences in patient demographics, procedure protocols, and clinical scale objectivity. It is not yet clear how different patient or procedural demographics impact treatment outcomes. As such, we chose to include studies despite the disparate range of populations and methodologies. We acknowledge, however, that the variability may limit generalizability. Yet, we believe that one of the values of this study is to have outlined the scope of this issue and demonstrated the need for standardized, multicenter studies in this field.

### 3.6 Strengths

The current metanalysis followed PRISMA guidelines and employed a systematic search across multiple databases. The use of random‐effects and mixed‐effects models for meta‐analysis and meta‐regression demonstrates strong statistical rigor. The recent other meta‐analyses used a random‐effects model only.

These results are novel because of the robustness of the methodological approach.

In addition, other meta‐analyses have limitations in their ability to assess treatment effect over time. Our meta‐analysis results overcome this limitation by exploring the durability of the MRgFUS treatment effect to a level of statistical significance, thereby providing further insight into the longevity of the reduction in hand tremor, a vital factor in a patient's desire to undergo this procedure.

## 4 Conclusions

The results of this meta‐analysis highlight the need to standardize the reporting of patient outcomes and AEs. This could be achieved with the use of digital, objective measures of the tremor to monitor the outcome of the surgery. In addition, there is a need to objectively monitor AEs such as gait and balance impairments, which would benefit from technological solutions. Attaining this aim will minimize heterogeneity across studies and will enhance our understanding of the benefits and limitations of the procedure.

In addition, our results showed that there is a lack of RCTs that assess long‐term efficacy. Indeed, there is only one published study reporting 5‐year follow‐up outcomes. We therefore suggest that there is a urgent need for extended follow‐up studies and longitudinal patient monitoring that focuses on patient‐centred outcomes as well as clinical efficacy.

Overall, this work provides a robust and up‐to‐date meta‐analysis of the treatment effect of MRgFUS in ET patients, including improvements in terms of tremor, disability, and QOL measures, in which effect sizes were significant. It also provides an analysis of AEs, including those reported intraoperatively and up to 1‐year post‐procedure. Our results suggest a decline in efficacy over time across 5 years, and a decline in the proportions of AEs across 1 year, but further evidence is needed to substantiate these findings. Future work could also investigate this intervention based upon patient characteristics such as SDR and cognitive impairment, target area, and unilateral versus bilateral procedures.

## Author Roles

(1) Research project: A. Conception, B. Organization, C. Execution; (2) Statistical Analysis: A. Design, B. Execution, C. Review and Critique; (3) Manuscript Preparation: A. Writing of the First Draft, B. Review and Critique.

A.S.: 1B, 1C, 2A, 2B, 3A.

S.L.: AC, 2A, 2B, 2C, 3B.

N.R.: 2C, 3B.

T.G.: 2C, 3B.

R.S.: 3B.

J.P.: 3B.

M.R.: 2C, 3B.

J.O.F.: 1A, 2C, 3B.

A.M.: 1A, 1B, 1C, 2A, 2C, 3B.

## Financial Disclosures

The authors declare that there are no additional disclosures to report.

## Supporting information


**Table S1.** Databases and the search strings used to identify relevant literature for this meta‐analysis.
**Table S2**. Total and hand tremor meta‐analysis effect sizes and heterogeneity metrics.
**Table S3**. Clinical Rating Scale for Tremor (CRST) Part C and Quality of Life in Essential Tremor Questionnaire scores (QUEST) meta‐analysis effect sizes and heterogeneity metrics.
**Table S4**. Adverse events proportions meta‐analysis effect sizes and heterogeneity metrics.
**Table S5**. Bilateral magnetic resonance‐guided focused ultrasound (MRgFUS) study patient and outcome measures.

**Figure S1.** Total Clinical Rating Scale for Tremor (CRST) Score forest plots. Forest plots depicting pre‐operation and post‐operation mean total tremor scores and their standard deviations for each study, standardized mean differences (SMDs), pooled SMDs, and heterogeneity metrics for total CRST scores 1 month (A), 3 months (B), 6 months (C), and 1 year (D) post‐operation.

**Figure S2.** Disability score forest plots. Displayed are the mean scores and standard deviations pre‐magnetic resonance‐guided focused ultrasound (MRgFUS) and post‐MRgFUS, standardized mean differences (SMDs), pooled SMDs, and measures of heterogeneity across studies reporting disability score changes from baseline to 1 month (A), 3 months (B), 6 months (C), and 1 year (D) postoperative.

**Figure S3.** Quality of Life in Essential Tremor Questionnaire scores (QUEST) score forest plots displaying the means, standard deviations, standardized mean differences (SMDs), pooled SMDs, and measures of heterogeneity across studies reporting QUEST score changes from baseline to 1 month (A), 3 months (B), 6 months (C), and 1 year (D) post‐operation.

**Figure S4.** Intraoperative adverse events (AEs) forest plots. Meta‐analysis results for the pooled proportions of intraoperative (intra‐op) AEs, displayed in forest plots for sensory events (A), nausea and vomiting (B), head pain (C), and dizziness (D). N&V, nausea and vomiting.

**Figure S5.** Forest plot of adverse events (AEs) at 1 month post‐operation. The pooled proportions and heterogeneity statistics of AEs at 1 month post‐operation, depicted in forest plots for sensory events (A), weakness (B), dysarthria (C), cerebellar events (D), and subjective cerebellar (E) events.

**Figure S6.** Forest plot of adverse events (AEs) 3 months post‐magnetic resonance‐guided focused ultrasound (MRgFUS). Forest plots displaying the pooled proportions and heterogeneity statistics of AEs at 3 months post‐MRgFUS, including cerebellar events (A), dysarthria (B), sensory events (C), subjective cerebellar events (D), and weakness (E).

**Figure S7.** Forest plot of adverse events (AEs) 6 months post‐magnetic resonance‐guided focused ultrasound (MRgFUS). Forest plots displaying the pooled proportions and heterogeneity statistics of AEs at 6 months post‐MRgFUS, including cerebellar events (A), sensory events (B), subjective cerebellar events (C), and weakness (D).

**Figure S8.** Forest plot of adverse events (AEs) 1 year post‐magnetic resonance‐guided focused ultrasound (MRgFUS). Forest plots displaying the pooled proportions and heterogeneity statistics of AEs at 1 year post‐MRgFUS, including sensory events (A), weakness (B), and cerebellar events (C).

## Data Availability

The data that support the findings of this study are available from the corresponding author upon reasonable request.
