# Supplementary material for: Efficacy and Safety of Magnetic Resonance‐Guided Focused Ultrasound Thalamotomy in Essential Tremor: A Systematic Review and Metanalysis
Source: Mov Disord. 2025 Apr 17;40(6):1020–33. doi: 10.1002/mds.30188 (PMC12160963; doi:10.1002/mds.30188)
Supplement: Supplementary file 1 — Table S1. Databases and the search strings used to identify relevant literature for this meta‐analysis. Table S2. Total and hand tremor meta‐analysis effect sizes and heterogeneity metrics. Table S3. Clinical Rating Scale for Tremor (CRST) Part C and Quality of Life in Essential Tremor Questionnaire scores (QUEST) meta‐analysis effect sizes and heterogeneity metrics. Table S4. Adverse events proportions meta‐analysis effect sizes and heterogeneity metrics. Table S5. Bilateral magnetic resonance‐guided focused ultrasound (MRgFUS) study patient and outcome measures. [file MDS-40-1020-s004.docx]

**Supplementary Table 1**

Supplementary Table 1: Databases and the search strings used to identify relevant literature for this meta-analysis.

| Database | Search string |
| --- | --- |
| PubMed | ("MRIgFUS"[Title/Abstract] OR "MRgFUS"[Title/Abstract] OR "focused ultrasound"[Title/Abstract] OR "HIFU"[Title/Abstract] OR ("ultrasound"[Title/Abstract] AND "thalamotomy"[Title/Abstract]) OR "high intensity focused ultrasound ablation"[MeSH Terms]) AND ("essential tremor"[Title/Abstract] OR "essential tremor"[MeSH Terms]) |
| Scopus | ( TITLE-ABS-KEY ( hifu ) OR TITLE-ABS-KEY ( "high intensity focused ultrasound ablation" ) OR TITLE-ABS-KEY ( "focused ultrasound" ) OR TITLE-ABS-KEY ( mrgfus ) OR TITLE-ABS-KEY ( mrigfus ) OR ( TITLE-ABS-KEY ( ultrasound ) AND TITLE-ABS-KEY ( thalamotomy ) ) ) AND ( TITLE-ABS-KEY ( essential AND tremor ) ) |
| Web of Science (WOS) | TS= (Essential tremor) AND TS= (HIFU OR high intensity focused ultrasound ablation OR focused ultrasound OR MRIgFUS OR MRgFUS OR (ultrasound AND thalamotomy)) |
| Cochrane library | ID Search  #1 MeSH descriptor: [Essential Tremor] this term only  #2 (essential tremor):ti,ab,kw  #3 MeSH descriptor: [High-Intensity Focused Ultrasound Ablation] this term only  #4 (focused ultrasound):ti,ab,kw  #5 (MRgFUS):ti,ab,kw  #6 (MRIgFUS):ti,ab,kw  #7 (HIFU):ti,ab,kw  #8 (ultrasound):ti,ab,kw  #9 (thalamotomy):ti,ab,kw  #10 #8 AND #9  #11 #10 OR #3 OR #4 OR #5 OR #6 OR #7  #12 #1 OR #2  #13 #11 AND #12 |

**Supplementary Table 2: Total and Hand Tremor Meta-Analysis Effect Sizes and Heterogeneity Metrics**

| **Outcome** | **SMD** | **Effect size P value** | **CI Lower** | **CI Upper** | **I^2^** | **Tau^2^** |
| --- | --- | --- | --- | --- | --- | --- |
| **Total CRST** |  |  |  |  |  |  |
| 1 month | -2.54 | < 0.0001 | -3.00 | -2.09 | 0.82 | 0.59 |
| 3 months | -1.87 | < 0.0001 | -2.22 | -1.53 | 0.68 | 0.25 |
| 6 months | -2.19 | < 0.0001 | -2.57 | -1.82 | 0.76 | 0.43 |
| 12 months | -2.08 | < 0.0001 | -2.52 | -1.63 | 0.78 | 0.42 |
| **Hand Score** |  |  |  |  |  |  |
| 1 month | -3.06 | < 0.0001 | -3.56 | -2.57 | 0.85 | 0.87 |
| 3 months | -2.35 | < 0.0001 | -2.81 | -1.90 | 0.79 | 0.39 |
| 6 months | -2.57 | < 0.0001 | -2.92 | -2.21 | 0.71 | 0.30 |
| 12 months | -2.36 | < 0.0001 | -2.84 | -1.89 | 0.76 | 0.40 |

Supplementary Table 2: A summary table of total and hand tremor outcome random-effects meta-analysis. SMD: Standardised mean difference. CI: Confidence interval.

**Supplementary Table 3: CRST Part C and QUEST Meta-Analysis Effect Sizes and Heterogeneity Metrics**

| **Outcome** | **SMD** | **Effect size P value** | **CI Lower** | **CI Upper** | **I^2^** | **Tau^2^** |
| --- | --- | --- | --- | --- | --- | --- |
| **Part C** |  |  |  |  |  |  |
| 1 month | -3.05 | < 0.0001 | -3.82 | -2.28 | 0.90 | 1.02 |
| 3 months | -2.35 | < 0.0001 | -2.91 | -1.79 | 0.82 | 0.52 |
| 6 months | -2.52 | < 0.0001 | -2.94 | -2.10 | 0.77 | 0.29 |
| 12 months | -2.85 | < 0.0001 | -4.04 | -1.67 | 0.87 | 2.24 |
| **QUEST** |  |  |  |  |  |  |
| 1 month | -1.47 | 0.0002 | -2.26 | -0.69 | 0.73 | 0.37 |
| 3 months | -1.46 | < 0.0001 | -2.11 | -0.82 | 0.51 | 0.17 |
| 6 months | -1.72 | < 0.0001 | -2.32 | -1.12 | 0.77 | 0.55 |
| 12 months | -1.41 | < 0.0001 | -1.92 | -0.90 | 0.54 | 0.15 |

Supplementary Table 3: A summary table of CRST Part C (Disability score) and QUEST outcome random-effects meta-analysis. SMD: Standardised mean differences. CI: Confidence interval.

**Supplementary Table 4: Adverse Events Proportions Meta-Analysis Effect Sizes and Heterogeneity Metrics**

| Timepoint | Category | Pooled Proportion [95% CI] | I^2^ | Tau^2^ |
| --- | --- | --- | --- | --- |
| Intra-operative | Sensory | 0.15 [0.05; 0.34] | 55.00% | 0.8405 |
|  | Dizziness | 0.33 [0.17; 0.54] | 74.60% | 0.9281 |
|  | Head pain | 0.39 [0.22; 0.59] | 71.90% | 0.8882 |
|  | Nausea & Vomiting | 0.29 [0.16; 0.47] | 66.50% | 0.6205 |
| 1 month | Sensory | 0.22 [0.15; 0.31] | 50.00% | 0.2203 |
|  | Weakness | 0.08 [0.05; 0.12] | 0.00% | < 0.0001 |
|  | Dysarthria | 0.09 [0.04; 0.17] | 14.40% | 0.0604 |
|  | Cerebellar | 0.21 [0.13; 0.31] | 58.50% | 0.4407 |
|  | Subjective Cerebellar | 0.23 [0.16; 0.31] | 30.80% | 0.0986 |
| 3 months | Sensory | 0.14 [0.08; 0.24] | 61.00% | 0.4181 |
|  | Weakness | 0.07 [0.04; 0.12] | 0.00% | 0.0000 |
|  | Dysarthria | 0.05 [0.02; 0.10] | 0.00% | 0.0000 |
|  | Cerebellar | 0.10 [0.06; 0.16] | 18.10% | 0.0974 |
|  | Subjective Cerebellar | 0.20 [0.15; 0.26] | 0.00% | 0.0000 |
| 6 months | Sensory | 0.19 [0.13; 0.27] | 46.90% | 0.1194 |
|  | Weakness | 0.04 [0.02; 0.08] | 0.00% | 0.0000 |
|  | Cerebellar | 0.09 [0.06; 0.14] | 0.00% | 0.0000 |
|  | Subjective Cerebellar | 0.13 [0.08; 0.19] | 0.00% | 0.0000 |
| 12 months | Sensory | 0.18 [0.13; 0.25] | 17.80% | 0.0491 |
|  | Weakness | 0.03 [0.01; 0.08] | 0.00% | 0.0000 |
|  | Cerebellar | 0.10 [0.04; 0.22] | 50.30% | 0.5505 |

Supplementary Table 4: A summary table of adverse event proportions meta-analysis results, including effect sizes, 95% confidence intervals and heterogeneity statistics.

**Supplementary Table 5: Bilateral MRgFUS Study, Patient and Outcome Measures**

| Author-Year | Iorio-Morin, 2021 | Martinez-Fernandez, 2021 |
| --- | --- | --- |
| Cohort size | 10 | 9 |
| Follow up | 3 months | 6 months |
| Age | 71.2 ± 7.5 | 71 ± 6 |
| Proportion male | 7M, 3F | 5M, 4F |
| Time since unilateral MRgFUS (months) | Median: 9 | Mean, SD: 24 ± 18 |
| SDR | Median: 0.42 (range: 0.35–0.71, IQR: 0.16) |  |
| Sonications no. | Median: 5.5 (range: 5–7, IQR: 1) |  |
| Peak temperature (̊C) | Median: 62 (range: 59–63, IQR: 3) Median maximal mean: 57 (range: 55–59, IQR: 2.4) |  |
| Total |  | Pre-op: 29.7 ± 11.5 6m: 15.5 ± 9.4 |
| HTS |  | Pre-op: 16.7 ± 2.9 6m: 5.9 ± 4.9 |
| Disability | Pre-op: 6.6 ± 3.7 3m: 2.4 ± 2.2 | Pre-op: 4.8 ± 4.6 6m: 3.1 ± 2.9 |
| QUEST | Pre-op: 35.1 ± 21.2 3m: 15.4 ± 11.3 |  |
| Adverse Events from second sided MRgFUS | 1m:  Subjective gait impairment (n=2), resolved by 3m  Falls (n=2), occurred within 1m post-op | Transient gait instability (n=5) Transient worsening of existing gait issues (n=1) Transient dysgeusia (n=1) Transient dysmetria (n=2) Transient worsening of existing dysarthria (n=1) Facial hypoesthesia (n=2) |
|  | 3m:  Slurred speech (n=1) Dysphagia (n=2) |  |
|  | Other: Motor neglect (n=2, 1 resolved by end of follow-up) Ataxia (n=5, all resolved) Dysgeusia (n=2, 1 resolved)  Dizziness (n=1, resolved) |  |

Supplementary Table 5: A summary table of study, patient and intervention characteristics, tremor and safety outcomes for bilateral MRgFUS studies. Pre-op: Before second sided thalamotomy.
