# Supplementary figures and images for: Efficacy and Safety of Magnetic Resonance‐Guided Focused Ultrasound Thalamotomy in Essential Tremor: A Systematic Review and Metanalysis
Source: Mov Disord. 2025 Apr 17;40(6):1020–33. doi: 10.1002/mds.30188 (PMC12160963; doi:10.1002/mds.30188)

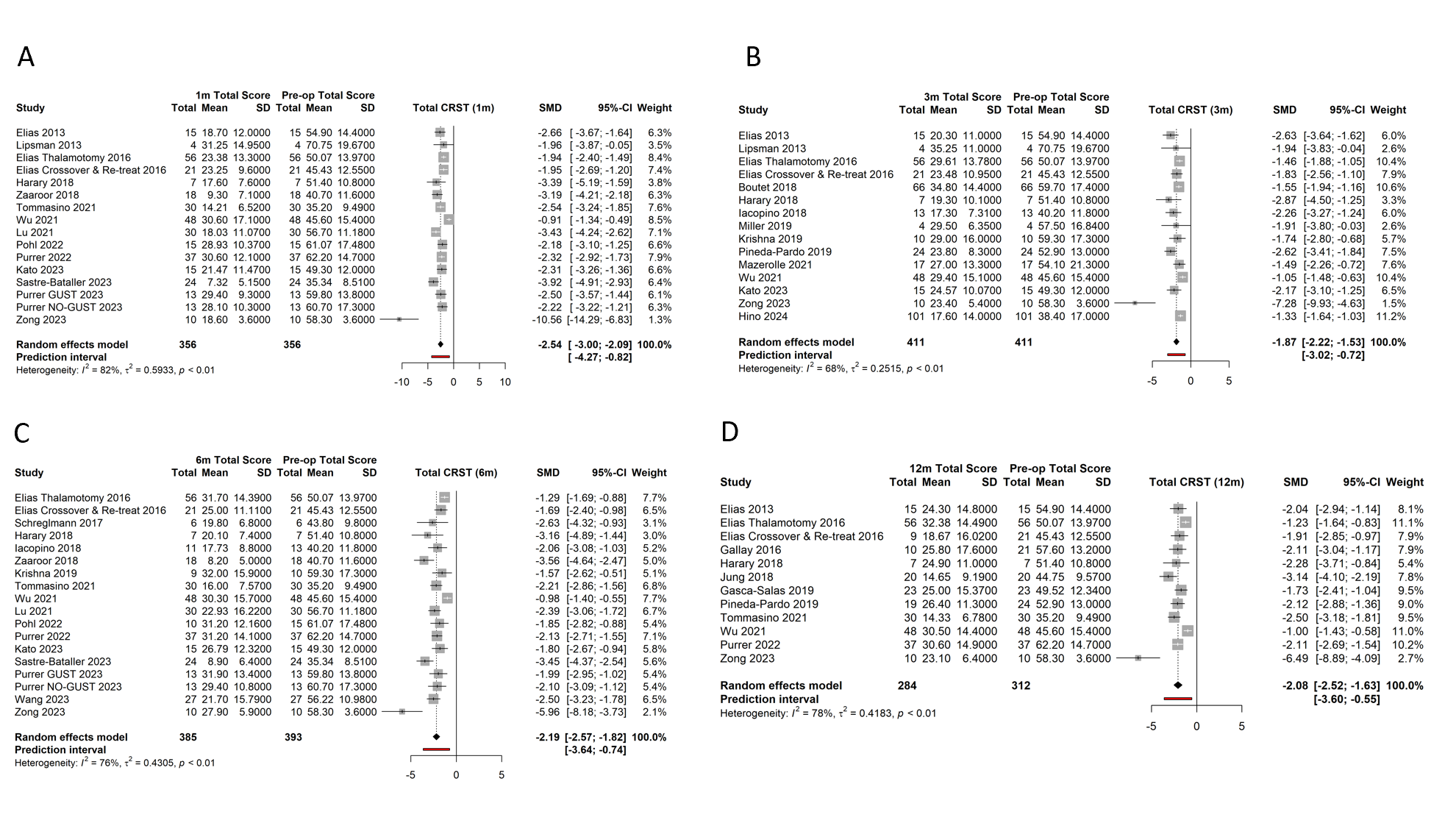

Supplement: Supplementary file 2 — Figure S1. Total Clinical Rating Scale for Tremor (CRST) Score forest plots. Forest plots depicting pre‐operation and post‐operation mean total tremor scores and their standard deviations for each study, standardized mean differences (SMDs), pooled SMDs, and heterogeneity metrics for total CRST scores 1 month (A), 3 months (B), 6 months (C), and 1 year (D) post‐operation. [file MDS-40-1020-s001.tiff]

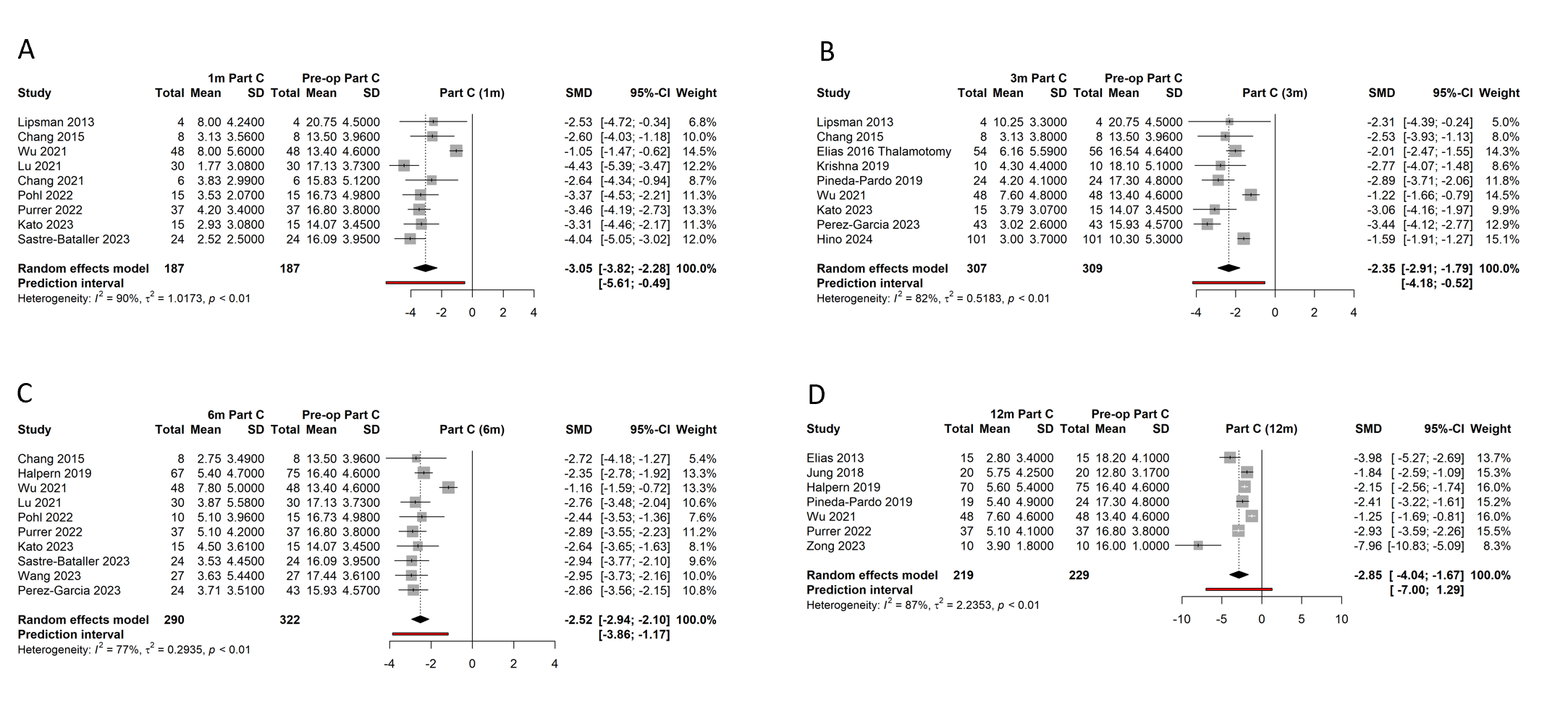

Supplement: Supplementary file 3 — Figure S2. Disability score forest plots. Displayed are the mean scores and standard deviations pre‐magnetic resonance‐guided focused ultrasound (MRgFUS) and post‐MRgFUS, standardized mean differences (SMDs), pooled SMDs, and measures of heterogeneity across studies reporting disability score changes from baseline to 1 month (A), 3 months (B), 6 months (C), and 1 year (D) postoperative. [file MDS-40-1020-s009.tiff]

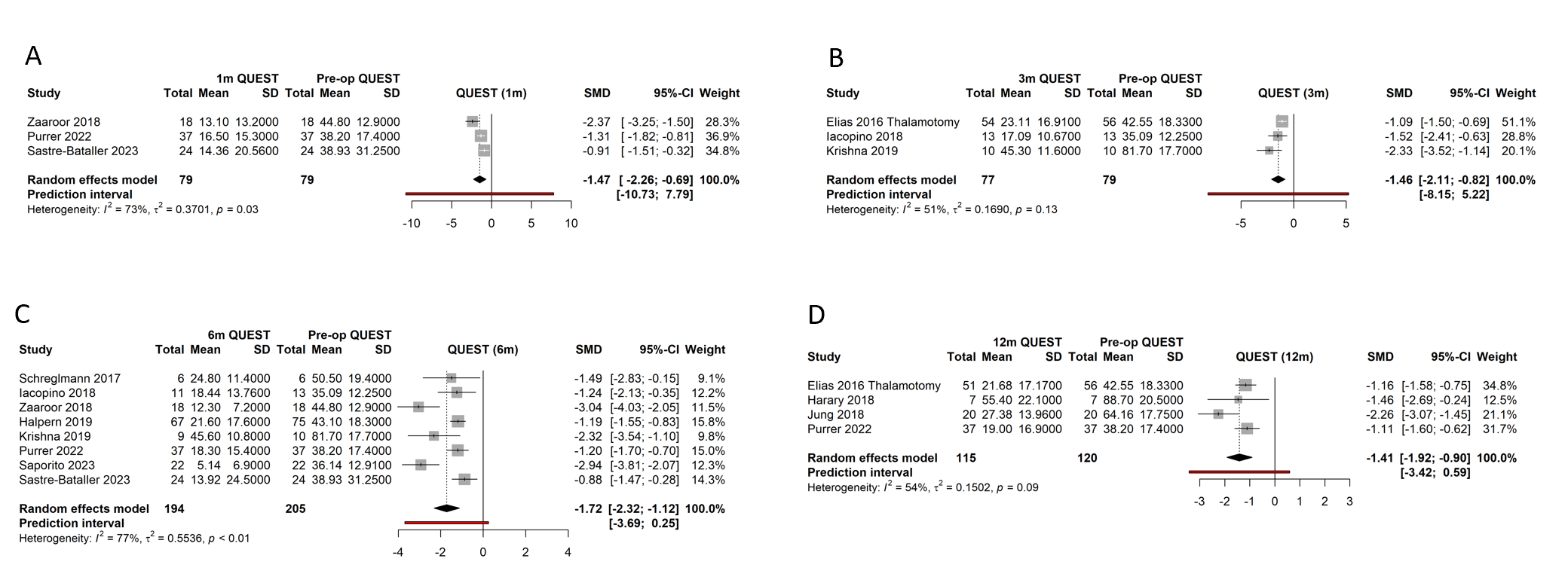

Supplement: Supplementary file 4 — Figure S3. Quality of Life in Essential Tremor Questionnaire scores (QUEST) score forest plots displaying the means, standard deviations, standardized mean differences (SMDs), pooled SMDs, and measures of heterogeneity across studies reporting QUEST score changes from baseline to 1 month (A), 3 months (B), 6 months (C), and 1 year (D) post‐operation. [file MDS-40-1020-s003.tiff]

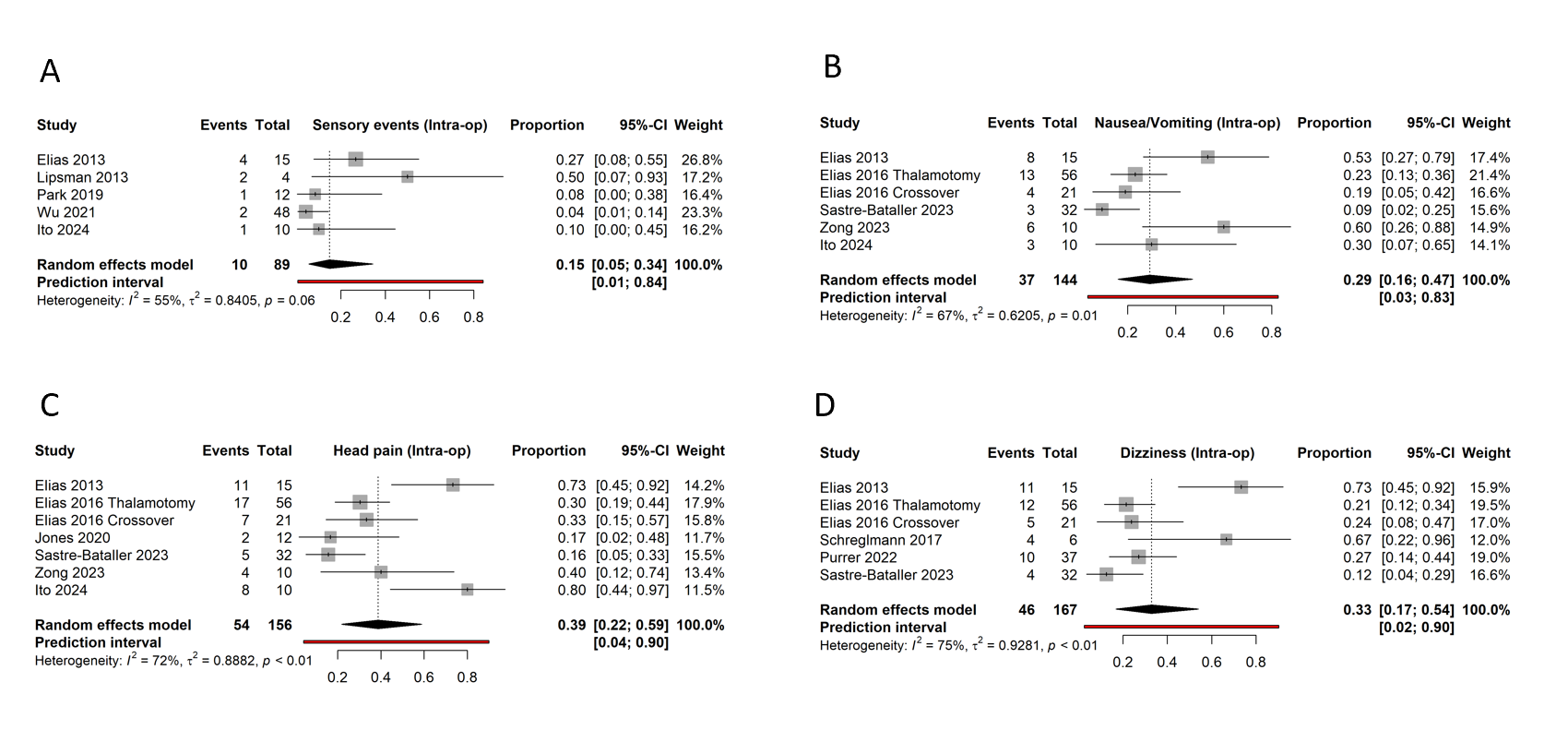

Supplement: Supplementary file 5 — Figure S4. Intraoperative adverse events (AEs) forest plots. Meta‐analysis results for the pooled proportions of intraoperative (intra‐op) AEs, displayed in forest plots for sensory events (A), nausea and vomiting (B), head pain (C), and dizziness (D). N&V, nausea and vomiting. [file MDS-40-1020-s007.tiff]

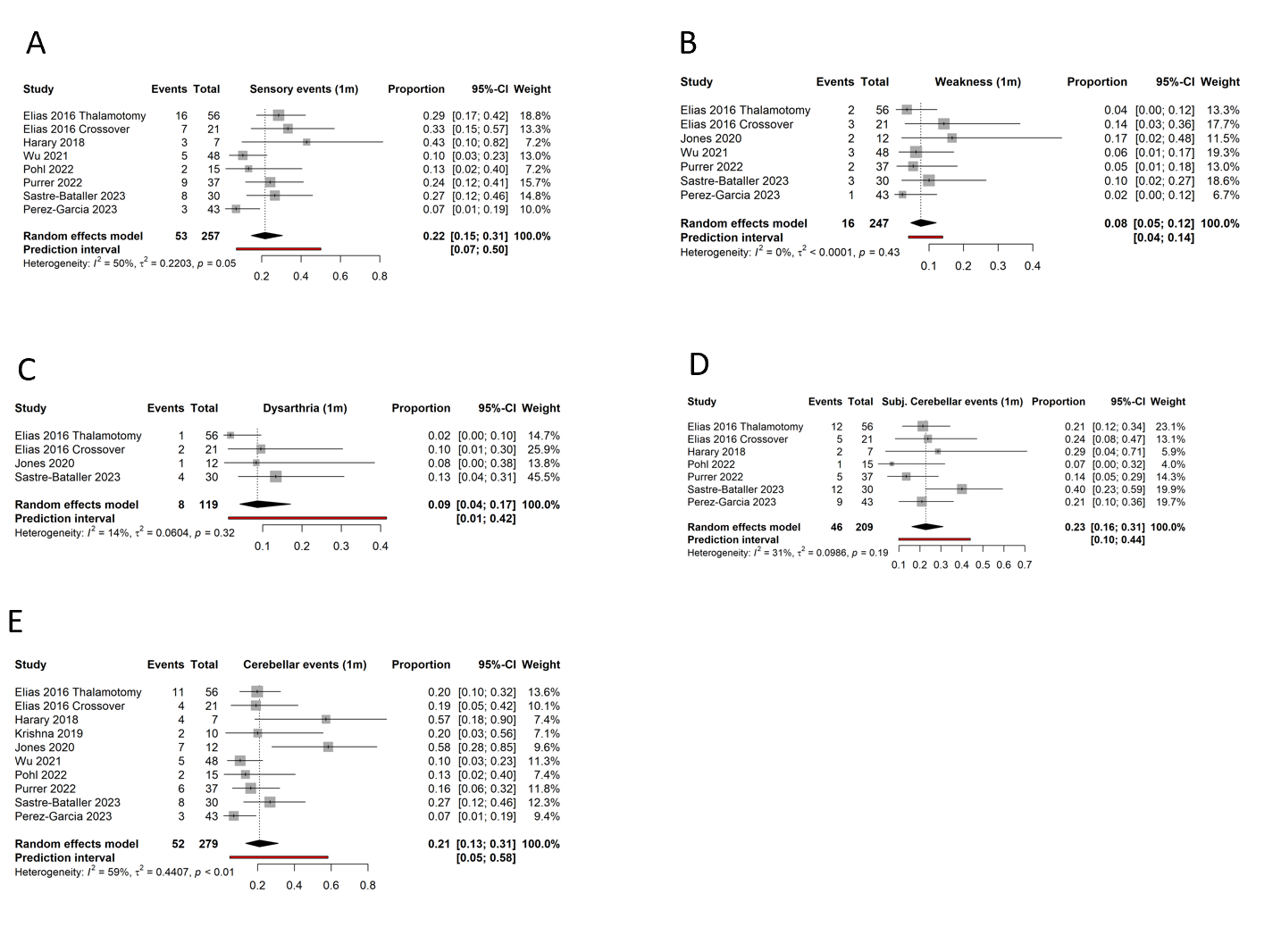

Supplement: Supplementary file 6 — Figure S5. Forest plot of adverse events (AEs) at 1 month post‐operation. The pooled proportions and heterogeneity statistics of AEs at 1 month post‐operation, depicted in forest plots for sensory events (A), weakness (B), dysarthria (C), cerebellar events (D), and subjective cerebellar (E) events. [file MDS-40-1020-s005.tiff]

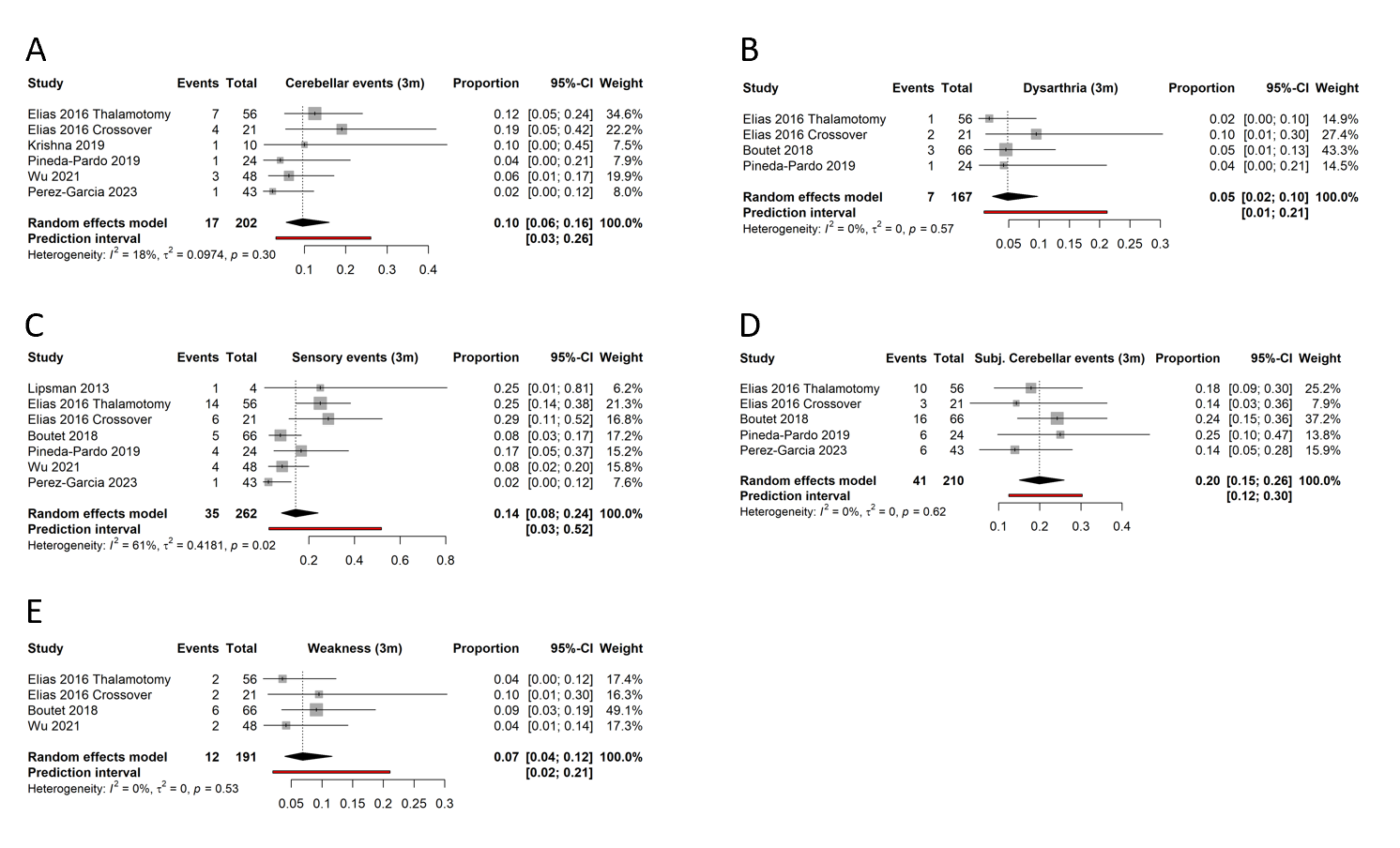

Supplement: Supplementary file 7 — Figure S6. Forest plot of adverse events (AEs) 3 months post‐magnetic resonance‐guided focused ultrasound (MRgFUS). Forest plots displaying the pooled proportions and heterogeneity statistics of AEs at 3 months post‐MRgFUS, including cerebellar events (A), dysarthria (B), sensory events (C), subjective cerebellar events (D), and weakness (E). [file MDS-40-1020-s002.tiff]

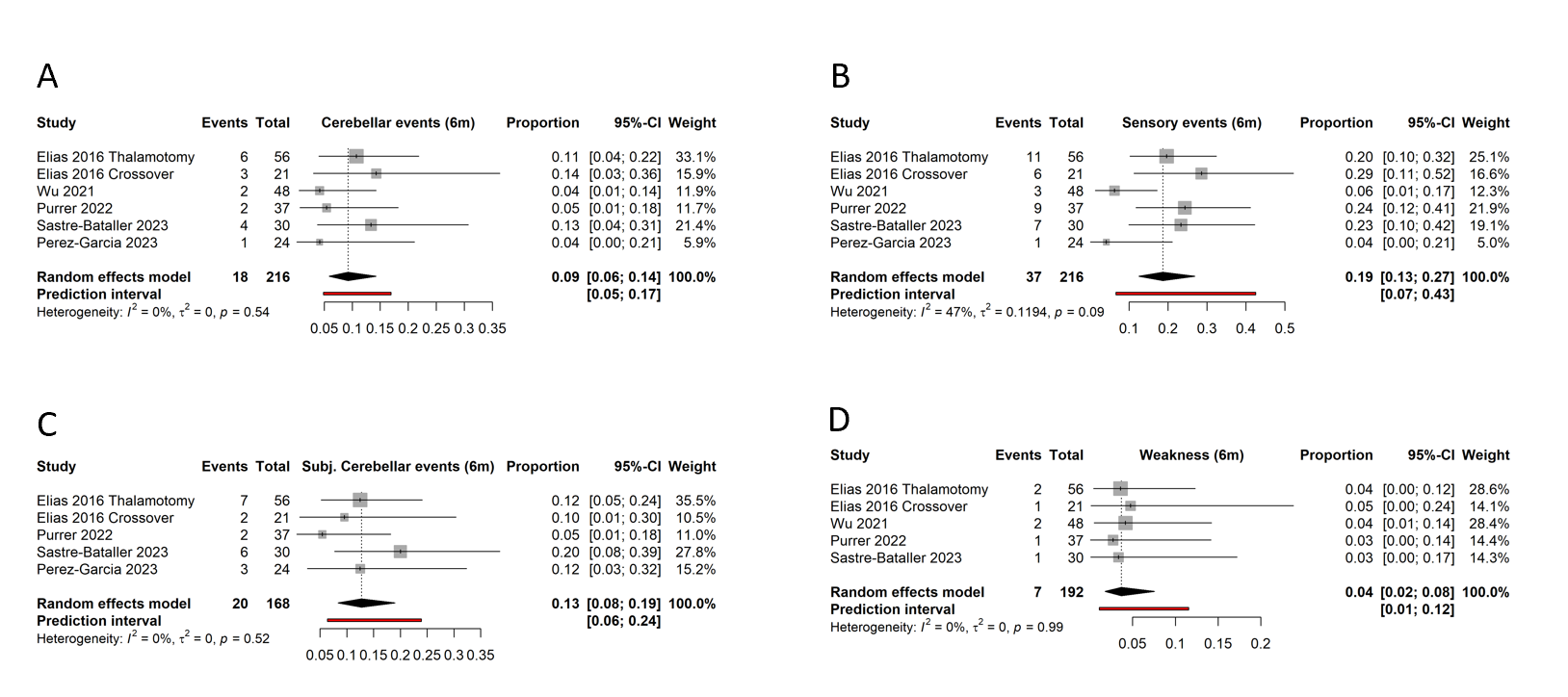

Supplement: Supplementary file 8 — Figure S7. Forest plot of adverse events (AEs) 6 months post‐magnetic resonance‐guided focused ultrasound (MRgFUS). Forest plots displaying the pooled proportions and heterogeneity statistics of AEs at 6 months post‐MRgFUS, including cerebellar events (A), sensory events (B), subjective cerebellar events (C), and weakness (D). [file MDS-40-1020-s008.tiff]

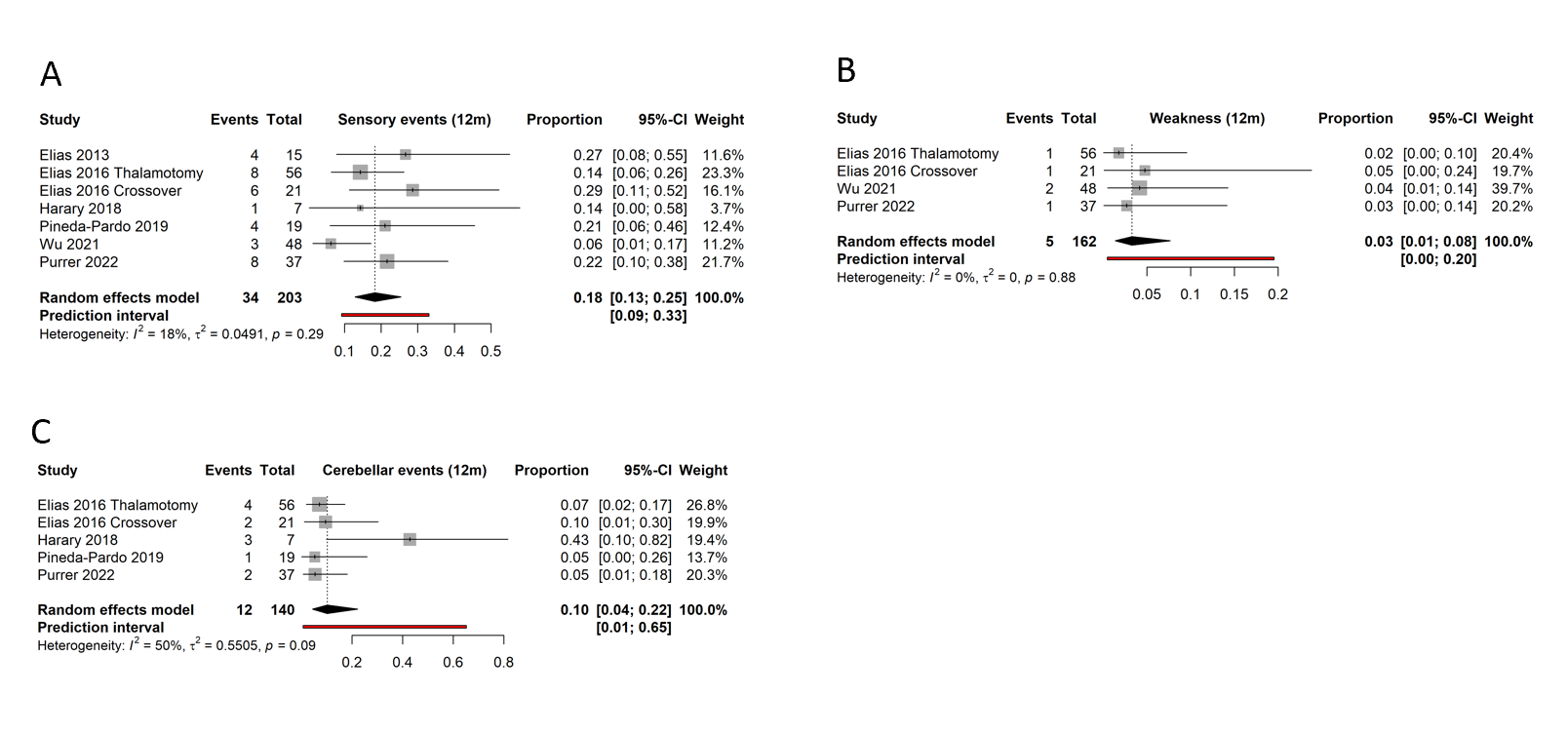

Supplement: Supplementary file 9 — Figure S8. Forest plot of adverse events (AEs) 1 year post‐magnetic resonance‐guided focused ultrasound (MRgFUS). Forest plots displaying the pooled proportions and heterogeneity statistics of AEs at 1 year post‐MRgFUS, including sensory events (A), weakness (B), and cerebellar events (C). [file MDS-40-1020-s006.tiff]
